# Multidisciplinary lifestyle intervention is associated with improvements in liver damage and in surrogate scores of NAFLD and liver fibrosis in morbidly obese patients

**DOI:** 10.1007/s00394-022-02846-7

**Published:** 2022-03-11

**Authors:** Monika Bischoff, Sebastian Zimny, Sebastian Feiner, Johannes Sauter, Svenja Sydor, Gerald Denk, Jutta M. Nagel, Gert Bischoff, Christian Rust, Simon Hohenester

**Affiliations:** 1 Krankenhaus Barmherzige Brüder München, Romanstrasse 93, 80639 Munich, Germany; 2 Department of Medicine II, LMU Munich, Marchioninistrasse 15, 81377 Munich, Germany; 3 Department of Internal Medicine, University Hospital Knappschaftskrankenhaus, Ruhr-University Bochum, In der Schornau 23-25, 44892 Bochum, Germany

**Keywords:** Morbid obesity, NAFLD, Lifestyle intervention, Liver fibrosis

## Abstract

**Purpose:**

Non-alcoholic fatty liver disease (NAFLD) is the hepatic manifestation of the metabolic syndrome. Particularly morbidly obese patients are at risk of developing progressive liver disease. Nutritional and lifestyle intervention is recommended as the standard of care in NAFLD. However, there is a striking lack of evidence to support the efficacy of lifestyle intervention to treat NAFLD in morbidly obese patients. Here, we aimed to assess the impact of lifestyle intervention on NAFLD in the morbidly obese in a real-world setting.

**Methods:**

136 obese patients were included in an industry-independent, multiprofessional lifestyle intervention program with a lead-in phase of 12 weeks of formula diet and a total of 48 weeks intensive counselling. Body weight and markers of the metabolic syndrome were analyzed. Presence of NAFLD was screened for by use of non-invasive markers of fatty liver, non-alcoholic steatohepatitis and liver fibrosis.

**Results:**

Weight loss goals (i.e. > 5% or > 10% of initial body weight, respectively, depending on baseline BMI) were achieved in 89.7% of subjects in the intention-to-treat analysis and 93.9% in the per-protocol analysis. This was associated with a pronounced improvement in serum ALT values. The percentage of subjects who fulfilled non-invasive criteria for fatty liver dropped from 95.2 to 54.8%. Risk of NASH improved and the number of patients at risk of liver fibrosis declined by 54.1%.

**Conclusion:**

Lifestyle intervention was associated with a marked improvement of serum ALT and an improvement of surrogate scores indicative of NAFLD and, importantly, advanced fibrosis, in a real-world cohort of morbidly obese patients.

**Supplementary Information:**

The online version contains supplementary material available at 10.1007/s00394-022-02846-7.

## Introduction

Obesity nowadays afflicts more than 40% of the population in industrialized countries, and its prevalence is still rising [1]. Obesity is the most important risk factor for developing the metabolic syndrome and its hepatic manifestation, i.e. non-alcoholic fatty liver disease (NAFLD). Thus, NAFLD has become the leading cause of chronic liver disease worldwide with an estimated prevalence of up to 30% [2, 3]. NAFLD encompasses a wide spectrum ranging from “benign” liver steatosis (non-alcoholic fatty liver, NAFL) to nonalcoholic steatohepatitis (NASH), which may progress to cirrhosis with its subsequent complications [2, 4] or lead to the development of hepatocellular carcinoma even in the absence of cirrhosis [5]. Thus, NAFLD has already become a major health burden, one which may become the major cause for liver transplantation in Western societies [4].

Large observational studies in the European population have found a four-fold higher NAFLD prevalence in obese than in lean patients [6, 7]. Moreover, a meta-analysis of eight observational studies demonstrated that obese patients had more severe histological lesions, including NASH and fibrosis [8], putting them at the highest risk of developing severe complications. Therefore, especially the obese are in need of effective therapies for NAFLD.

Despite great efforts, no medical therapies for NAFLD have been approved so far. Because of the close link between obesity and NAFLD, weight reduction through lifestyle intervention is recommended in current guidelines [9]. However, only few studies have shown that weight loss achieved by lifestyle intervention is effective in treating NAFLD. Furthermore, trials investigating into the efficacy of lifestyle intervention for the treatment of NAFLD were almost exclusively performed in overweight patients or patients with class I obesity only (max. BMI 35 kg/m^2^) [10–13]. Thus, there is a lack of data assessing the efficacy of lifestyle intervention for NAFLD in morbidly obese patients, although this group of patients is at highest risk of progressive liver disease. Furthermore, despite clear recommendations in current guidelines [9], the efficacy of lifestyle intervention programs in treating NAFLD and NASH is still contested [14]. More data to assess their efficacy with regard to NAFLD, especially in the very obese, are urgently needed.

The gold standard to assess NAFLD is liver histology. However, this is only feasible in clinical studies, which are hampered by selection bias and place enrollment hurdles that are not easily overcome by many patients. Thus, data obtained in a “real-world” setting are needed, more reflective of a broader patient spectrum and allowing for extrapolation into day-to-day clinical routine. Owing to the observational design of our study, non-invasive markers to monitor NAFLD were used.

It was our aim to investigate into the efficacy of multidisciplinary lifestyle intervention in targeting NAFLD in patients with morbid obesity in a real-life setting. Indicators of obesity, the metabolic syndrome, NAFLD, liver fibrosis and NASH were analyzed. Non-invasive markers were used to assess NAFLD, as recommended by the European Association for the Study of the Liver (EASL) [9, 15]. We analyzed (i) liver steatosis (fatty liver index (FLI) [16]), (ii) liver damage (ALT, applying revisited range values for NAFLD patients [9, 15, 17]) (iii) liver fibrosis (NAFLD fibrosis score (NFS) [18]) and NASH, the latter non-invasively defined by serum levels of M30 [19] or lipidome analysis.

## Methods

### Selection of patients

Obese patients (BMI > 30 kg/m^2^) aged > 18 years were screened for participation in our lifestyle intervention program as part of their standard clinical care. Exclusion criteria for participation in the program were the presence of malignant disease, presence of other chronic liver disease including viral hepatitis, pregnancy, alcohol consumption (> 10 g per day for females and > 20 g per day for males, respectively), other addictions or instable psychiatric disease. Patients were included in a data- and biobank after informed consent was obtained. The study was approved by the Ethics Committee at the Medical Faculty of the University of Munich (protocol 18–358).

### Multidisciplinary lifestyle intervention program

Our lifestyle intervention program recognizes obesity as a chronic disease. It is set up as a multimodal and multiprofessional, industry-independent program for the sustained treatment of obesity and is designed to treat and follow the patients for 12 months. This so-called “ZEPmax”-program is based at the “Zentrum für Ernährungsmedizin und Prävention” (ZEP, Center for Nutritional Medicine and Prevention) in Munich, Germany.

Patients were assigned to closed groups, with 14 adults per group on average. Group sessions were held weekly for 3.5 h. In total, 34 units of behavior therapy and 30 units of dietary counseling in individual and group sessions were conducted, in addition to 2 cooking classes, 50 units of group exercise therapy and 14 physician appointments. The program was divided into 3 phases: 12 weeks formula diet to induce weight loss, 3 weeks of adaptation to a hypocaloric regular diet and 35 weeks of regular diet implementing recommendations for a healthy diet. Within the first 12 weeks, formula diet was provided as the sole source of nourishment. Four servings of formula diet per day were balanced to 859 kcal, containing 55 g protein, 109 g carbohydrates and 20 g fat (41.3% saturated fatty acids, 14.2% mono-unsaturated fatty acids, 22.3% di- and polyunsaturated fatty acids and 22.2% linolic acid). Formula diet was purchased from Wander AG (Neuenegg, Switzerland). Composition of the formula differed only slightly between different flavors of formula (max. 3% difference in daily calorie intake). During the adaptation phase, formula diet was reduced stepwise and replaced by a hypocaloric, self-prepared diet rich in protein. In the following 35 weeks, the focus laid on weight stabilization with a self-prepared calorie-reduced mixed diet accompanied by intensive nutritional advice, behavior therapy, and exercise therapy.

Regarding alcohol consumption, patients with moderate alcohol use at screening were asked to omit alcohol consumption during the formula diet phase and were advised to limit alcohol consumption as part of their dietary counselling.

As recommended by current guidelines, weight loss was categorized as successful (goal achieved) if participants with a baseline BMI of < 35 kg/m^2^ lost > 5% and participants with a baseline BMI of  ≥ 35 kg/m^2^ lost > 10% body weight, respectively.

### Assessment of glucose metabolism, liver steatosis and liver fibrosis

Impaired fasting glucose (≥ 100 mg/dL) and diabetes (≥ 126 mg/dL) were defined in accordance with current guidelines [20]. Patients on antidiabetic medication were considered diabetic irrespective of their fasting glucose levels.

In assessing NAFLD-associated liver damage, adjusted ALT range values (men: < 30 U/L, women: < 19 U/L) were applied as recommended by EASL [15].

The FLI is an established scoring system, predicting liver steatosis with high accuracy [16]. FLI was calculated from BMI, waist circumference, triglycerides and gamma-GT to rule out (FLI < 30) or rule in (FLI ≥ 60) fatty liver. The NFS is a validated score, allowing the discrimination between patients with and without advanced fibrosis with high accuracy [18]. NFS was calculated from age, BMI, ALT, blood platelet count, serum albumin and presence or absence of impaired fasting glucose. Both FLI and NFS are recommended by EASL as tools to evaluate NAFLD [9, 15].

In addition, the ‘fibrosis improvement during lifestyle intervention’ (FILI) score was applied to assess improvement in NAFLD [21]. This score was developed from a lifestyle intervention study with paired liver biopsies in 261 NASH patients and includes changes in HbA1c, platelet number and ALT normalization. The FILI score discriminated patients with fibrosis improvement significantly better than other biomarkers including NFS.

### M30 measurement

For the assessment of cell death due to apoptosis, the biomarker M30 was quantified in patients’ sera by ELISA (Apoptosense®, PEVIVA, Alexis) according to the manufacturer's instructions. Measurements were done in one batch at the end of the study.

### Lipidomics

At day 0 and week 48, fatty acid levels and fatty acid composition were determined in serum. Extraction from serum was performed according to the method of Folch with chloroform/methanol (2:1 v/v) [22]. The extract was washed once with NaCl (0.9%, 0.2-fold volume) and twice with methanol/water (1:1 v/v). Subsequently, the organic phase was evaporated via N_2_, esterified with derivatization reagent (Supelco®, Germany) and resolved in n-hexane (hexane/water, 1:1). Fatty acid methyl esters were separated by capillary gas chromatography (BPX70 column from SGE®, Germany, oven temperature 120–210 °C with increase of 2 °C/min, H_2_ as carrier gas, flame ionization detector) and identified by comparison of their retention time relative to those of the standard mixture (37 component FAME mix, Supelco®, Germany). Quantification was performed with C15:0 as internal standard by use of the Clarity Lite software (DataApex).

### Statistics

All linear data are presented as mean ± standard deviation (SD). Statistics were performed with SPSS 25. For the intention-to-treat analysis, the last observation was carried forward. A linear model of repeated measure was applied (repeated measurement ANOVA) and multiple paired tests were adjusted with Bonferroni correction. Statistics for nominal data were calculated with Pearson's Chi-squared test. For paired nominal data, the McNemar test was applied.

## Results

### Treatment response was achieved in a majority of patients

**Fig. 1:**
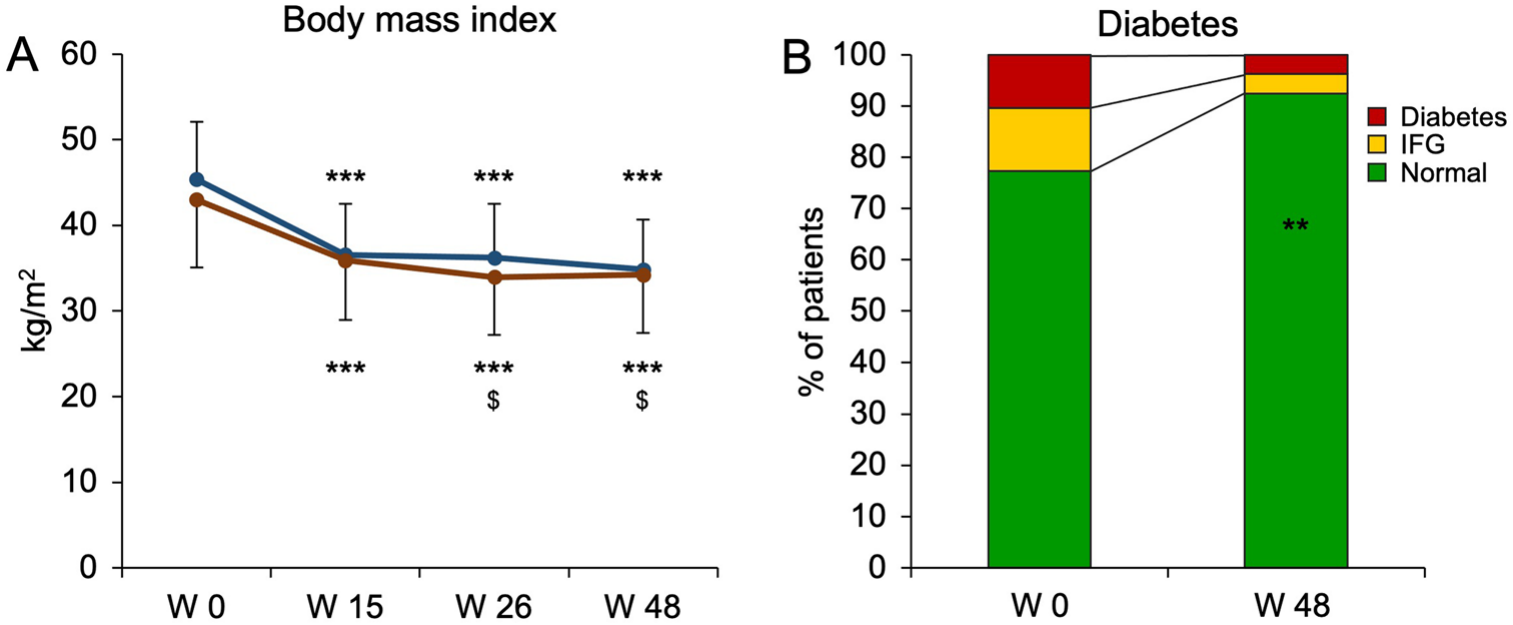
Body weight and glycemic status improve with lifestyle intervention. A total of 136 obese subjects were included in the lifestyle intervention program. 114 (84%) participants could be included in the per protocol analysis. **A** BMI is given over time for males (blue) and females (red) showing marked and sustained weight loss. ***p < 0.001 vs. W0, ^$^*p* < 0.001 vs. W15, ANOVA, Bonferroni-adjusted post-hoc analysis. **B** Glycemic status was followed over time. Subjects on antidiabetic medication or fasting plasma glucose levels ≥ 126 mg/dL were considered diabetic (red), while subjects with fasting plasma glucose levels 100–125 mg/dL were defined as subjects with impaired fasting glucose (IFG). As a result of lifestyle intervention, glycemic status of patients markedly improved. **p < 0.01, McNemar test

A total of 145 subjects were screened and 136 subjects participated in our multimodal lifestyle intervention program. A flowchart of the study population is given in supplementary Fig. 1. In the intention-to-treat analysis with last observation carried forward, 122/136 (89.7%) subjects achieved their weight loss goals (i.e. patients lost > 5% and > 10% of weight depending on the baseline BMI of < 35 and ≥ 35 kg/m^2^, respectively). Intervention was completed by 114 patients (per protocol cohort). Baseline characteristics of this cohort stratified for gender are given in Table 1 (additional variables and stratification for obesity class are shown in supplementary Table 1). Almost three quarters of participants were female. All participants were obese (BMI ≥ 30 kg/m^2^) and 65.8% were morbidly obese with a BMI > 40 kg/m^2^. Weight loss goals were achieved by 107/114 (93.9%). The weight loss over time is shown in Fig. 1A. At the start of the program, participants had a significant initial drop in body weight during the formula diet phase. Importantly, the initial weight loss was maintained during the re-introduction of a regular diet both in the initial hypocaloric phase as well as in the maintenance phase, in which participants prepared their own food and implemented lifestyle changes to keep a stable steady state. Weight cycling or weight gain could be avoided over the long-term observational period of 48 weeks.

**Table 1:** Participants’ characteristics

	Total	Male	Female	*p*
Sex, *n* (%)	114 (100%)	31 (27.2%)	83 (72.8%)	n.a
Age (years)	45.4 ± 13.3	48.7 ± 11.7	44.2 ± 13.6	n.s
Weight (kg)	128.3 ± 26.4	146.6 ± 25.8	121.5 ± 23.3	< 0.001 ^§^
BMI (kg/m^2^)	43.6 ± 7.7	45.4 ± 6.7	43.0 ± 8.0	n.s
Obesity class I (BMI ≥ 30), *n* (%)	13 (11.4%)	1 (3.2%)	12 (14.5%)	n.s
Obesity class II (BMI ≥ 35), *n* (%)	26 (22.8%)	7 (22.6%)	19 (22.9%)	
Obesity class III (BMI ≥ 40), *n* (%)	75 (65.8%)	23 (74.2%)	52 (62.7%)	
Treatment response, *n* (%)	107 (93.9%)	29 (93.5%)	78 (94.0%)	n.s
Relative weight loss (%)	21.0 ± 8.6	22.9 ± .9.7	20.3 ± 8.0	n.s
Weight loss < 10%, *n* (%)	8 (7.0%)	2 (6.5%)	6 (7.2%)	0.09^$^
Weight loss 10–20%, *n* (%)	44 (38.6%)	7 (22.6%)	37 (44.6%)	
Weight loss 20–30%, *n* (%)	45 (39.5%)	14 (45.2%)	31 (37.3%)	
Weight loss > 30%, *n* (%)	17 (14.9%)	8 (25.8%)	9 (10.8%)	

A total of 136 obese patients were included in the lifestyle intervention program. Baseline characteristics are given for the cohort in the per protocol analysis (*n* = 114). The majority of patients was female. Treatment response was defined as weight loss of > 5% and > 10% of body weight depending on a baseline BMI of < 35 and ≥ 35 kg/m^2^, respectively. *p* values were calculated for the comparison of male and female participants applying ^§^ unpaired *t* test and ^$^ Chi-squared test, respectively

In summary, our multimodal lifestyle intervention enrolled a real-world cohort comprised of mainly morbidly obese subjects, with low drop-out rates. Participants achieved and sustained a substantial weight loss during the entire course of 48 weeks.

### Weight loss and lifestyle change were associated with an improvement in components of the metabolic syndrome

NAFLD is the hepatic manifestation of the metabolic syndrome. Components of the metabolic syndrome are, among others, diabetes and elevated serum triglycerides. The proportion of subjects with impaired glucose tolerance or diabetes declined from 22.8% at baseline to 7.5% at the end of the program (*p* < 0.01, McNemar-test; Fig. 1B). Serum cholesterol (200.2 mg/dL ± 45.2 mg/dL vs. 186.5 mg/dL ± 38.2 mg/dL, *p* < 0.001, *t* test) and serum triglycerides (138.9 mg/dL ± 55.8 mg/dL vs. 105.3 mg/dL ± 53.3 mg/dL, *p* < 0.001, *t* test) also improved. As a surrogate for insulin sensitivity, the triglycerides/HDL ratio was calculated and significantly improved from 2.7 ± 1.4 to 1.8 ± 1.2 (*p* < 0.001, *t* test). No patient was newly prescribed with anti-diabetic or lipid lowering medication during the course of the program.

Thus, as expected, weight loss was associated with improvements in components of the metabolic syndrome.

### Lifestyle intervention is associated with specific changes in the serum lipidome

In experimental NASH, specific alterations of the lipidome have been described [23, 24]. In patients, the composition of the hepatic [25] and serum [26, 27] lipidome has repeatedly been associated with NAFLD, and specific alterations in fatty acid composition were found to correlate with (histology-proven) NASH. Experimentally, fatty acid-induced alterations of mitochondrial function have been suggested as a potential underlying mechanism [28]. Therefore, 21 individual fatty acids were quantified in participants´ sera at enrollment and at 48 weeks (supplementary Table 2). Results demonstrate a relatively stable pattern of fatty acid composition. While serum levels of the polyunsaturated omega-9 fatty acid C20:1n9 (eicosenoic acid) were increased, levels of the monounsaturated fatty acid C16:1n7 (palmitoleic acid) dropped after weight loss. Relevant fatty acid ratios and components are given in Table 2.

**Table 2:** Change in fatty acid composition during lifestyle intervention

	W0	W48	*p*
Total FA	18.6 g/l ± 9.5 g/l	20.0 g/l ± 14.0 g/l	0.317
Saturated FA	8.3 g/l ± 3.7 g/l	8.3 g/l ± 3.9 g/l	0.903
Unsaturated FA	10.3 g/l ± 6.7 g/l	11.7 g/l ± 11.3 g/l	0.271
MUFA	6.0 g/l ± 5.5 g/l	7.4 g/l ± 10.7 g/l	0.256
DUFA	3.1 g/l ± 1.5 g/l	3.1 g/l ± 1.7 g/l	0.886
PUFA	1.3 g/l ± 0.8 g/l	1.2 g/l ± 0.8 g/l	0.364
Saturated to unsaturated	0.9 ± 0.4	0.9 ± 0.4	0.707
C16:1n7 to C16:0	0.05 ± 0.02	0.04 ± 0.02	** < 0.001**
C18:0 to C16:0	0.6 ± 0.2	0.6 ± 0.2	0.434

Lipidomics were determined by gas chromatography. Total, saturated and unsaturated fatty acids (FA) are shown, as well as mono-unsaturated (MUFA), di-unsaturated (DUFA) and poly-unsaturated (PUFA) fatty acids and relevant fatty acid ratios. Weight loss was associated with changes in the C16:1n7 to C16:0 ratio. *p* values are given for paired *t* tests

Overall, rather than general changes, weight loss and change of lifestyle led to specific changes in fatty acid composition, namely a decrease in the C16:1n7 to C16:0 ratio.

### Weight loss and lifestyle changes were associated with amelioration of liver damage and risk of liver steatosis

**Fig. 2:**
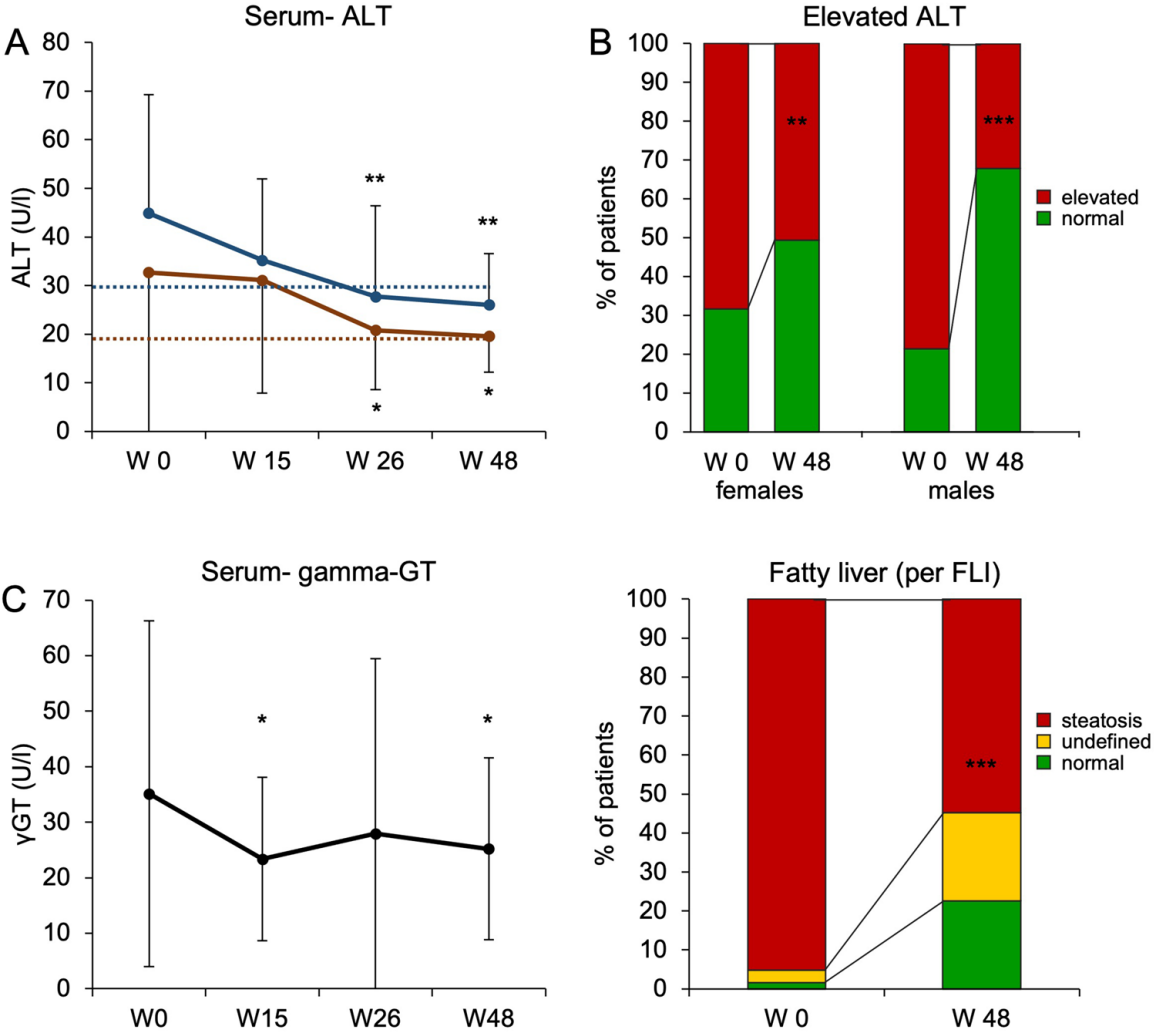
Liver damage and risk of fatty liver improve with lifestyle intervention A total of 136 obese subjects were included in the lifestyle intervention program. 114 (84%) subjects could be included in the per protocol analysis. **A** Serum ALT values over time are given for female (red) and male (blue) participants, respectively. Dotted lines mark the gender-specific upper limits of normal (ULN) at 19U/L (females) and 30U/L (males). *p < 0.05, **p < 0.01 vs. W0, ANOVA, Bonferroni-adjusted post-hoc analysis. **B** The percentage of subjects with elevated (red) or normal ALT values (green) before and after lifestyle intervention is given, accounting for gender-specific ULN. **p < 0.01, ***p < 0.001, McNemar test. **C** γGT values are given over time. *p < 0.05 vs. W0, ANOVA, Bonferroni-adjusted post-hoc analysis. **D** Fatty liver was assessed by calculation of FLI. Subjects in which fatty liver was excluded are depicted in green, subjects with indeterminate values are shown in yellow and those with definite fatty liver are shown in red. Weight loss during lifestyle intervention was associated with a marked decrease in the proportion of patients with fatty liver. ***p < 0.001, Chi-squared test

We determined serum levels of ALT and gamma-GT during the course of the multimodal lifestyle intervention program. To this aim, we applied the published revisited range ALT values for NAFLD patients (with values > 30U/L for males and > 19U/L for females being considered elevated) [9, 15, 17]. There was a pronounced and continuous decline in serum ALT activity over the entire course of the program (Fig. 2A). The prevalence of elevated serum ALT levels dropped from 70.1% at baseline to 45.8% at the end of the program. Improvement of serum ALT levels showed a very weak correlation with change of body weight (*p* = 0.02, *r*^2^ = 0.07) as depicted in supplementary Fig. 2. In males, the proportion of participants with elevated ALT fell from 78.6 to 32.1% (females: 68.4% vs. 50.6%, Fig. 2B). There also was a significant decline of gamma-GT levels over time (Fig. 2C). Levels of gamma-GT, however, did not follow the same pattern over time as ALT levels.

As depicted in Fig. 2D, weight loss and lifestyle changes were associated with a marked decline in the risk of fatty liver as defined by FLI. While the percentage of subjects with NAFL declined from 95.2 to 54.8%, liver steatosis could be ruled out in almost a quarter of all subjects (22.6% vs. 1.6%) after participation in lifestyle intervention (*p* < 0.001, Chi-squared test).

In summary, liver damage as assessed by serum ALT markedly improved in our cohort of morbidly obese subjects during long-term lifestyle intervention. Weight loss was furthermore associated with a decreased risk for NAFL.

### M30 levels and liver fibrosis are ameliorated in association with lifestyle intervention

**Fig. 3:**
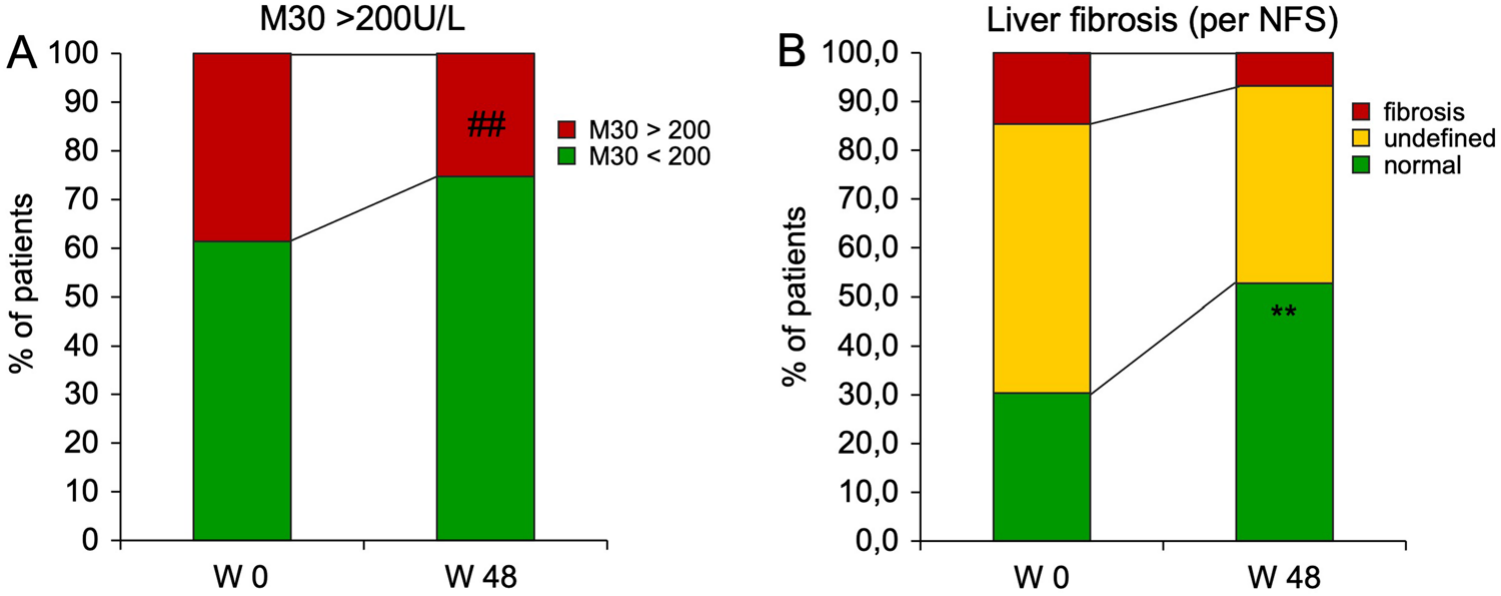
Serum M30 values and risk of liver fibrosis improve with weight loss during lifestyle intervention. **A** As a surrogate for NASH, patients were classified per M30 values with a cut-off set at 200U/L. Weight loss led to a decrease in participants with M30 values > 200U/L. ^##^*p* < 0.01, McNemar. **B** NFS was calculated and participants in whom liver fibrosis could be excluded are depicted in green, participants with indeterminate values are shown in yellow and those with definite fibrosis are shown in red. Weight loss during lifestyle intervention was associated with a marked decrease in the proportion of subjects at risk for liver fibrosis. **p < 0.01, Chi-squared test

The serum apoptosis marker M30 has been repeatedly used to non-invasively identify patients at increased risk of NASH or advanced liver disease, using a cut-off of 200U/L [19, 29–31]. We therefore determined serum M30 values in our patients over the time course of the study. At enrollment, M30 values > 200U/L were detected in 38.6% of participants. After weight loss and lifestyle change, the percentage of participants with M30 > 200U/L declined to 25.3% (*p* < 0.01, Fig. 3A). Absolute M30 values declined moderately but highly significantly from 206.4 U/L ± 168.0 U/L to 167.3 U/L ± 147.7 U/L (*p* < 0.001, paired *t* test). M30 correlated considerably with ALT (*r* = 0.55, *p* < 0.001, Spearman rank) but weakly with body weight (*r* = 0.25, *p* = 0.02, Spearman rank).

The fatty acid ratio C16:1n7/C16:0 in serum has been reported to closely correlate with the histological presence of NASH [27]. As described above, the C16:1n7/C16:0 ratio specifically and highly significantly declined by 20% in our cohort, whereas the further fatty acid profile remained unchanged.

Even more than the presence of NASH, NAFLD-associated liver fibrosis is prognostic for liver-related morbidity and mortality [32]. NFS was used to identify subjects with liver fibrosis [9, 15]. Its cut-off values rule in and rule out liver fibrosis. During lifestyle intervention, the number of participants with a high likelihood for liver fibrosis was reduced by more than half (absolute proportions fell from 14.6 to 6.7%, *p* < 0.01, Chi-squared test, Fig. 3B). Conversely, liver fibrosis could be ruled out in 52.8% of participants after lifestyle intervention (vs. 30.3% before intervention). Positive changes in terms of liver fibrosis were further supported by the FILI score [21]. In line with the results determined by NFS, FILI indicated an improvement of liver fibrosis in 43.2% of our subjects. A summary of key anthropometric, metabolic and hepatic variables and their change over time is given in Table 3.

**Table 3:** Summary of key antoprometric, metabolic and hepatic variables

Parameter	W0	W48	*p*
Weight (kg)	128.3 ± 26.4	101.0 ± 21.8	< 0.01
Waist (cm)	126.5 ± 16.9	102.3 ± 14.8	< 0.01
GOT (U/L)	28.4 ± 21.2	22.6 ± 7.0	< 0.01
GPT (U/L)	35.9 ± 32.4	21.4 ± 8.8	< 0.01
gGT (U/L)	35.1 ± 31.2	25.2 ± 16.4	< 0.01
Cholesterol (mg/dL)	200.2 ± 45.2	186.5 ± 38.2	< 0.01
Triglycerides (mg/dL)	138.9 ± 55.8	105.3 ± 53.3	< 0.01
Glucose (mg/dL)	94.3 ± 29.9	81.7 ± 12.9	< 0.01
M30 (U/L)	206.4 ± 168.0	167.3 ± 147.7	< 0.01
FLI	93.8 ± 12.3	62.6 ± 29.3	< 0.01
NFS	− 0.8 ± 1.6	− 1.6 ± 1.3	< 0.01

A summary of key antoprometric, metabolic and hepatic variables is given for the per protocol cohort (*n* = 114) and changes over time are given. *p* values were calculated in paired analysis (*t* tests)

In summary, weight loss and lifestyle changes were associated with significantly reduced likelihoods of both NASH and liver fibrosis, each determined by two independent non-invasive measures.

## Discussion

In our observational study, reflecting a real-world setting, we demonstrated excellent therapeutic response in terms of weight loss in an industry-independent 48-week multidisciplinary lifestyle intervention program in morbidly obese patients. Lifestyle intervention was furthermore associated with marked improvement in surrogate markers of non-alcoholic fatty liver disease. Thus, our results support the efficacy of weight loss by lifestyle intervention to treat NAFLD in patients with morbid obesity.

Although it is generally recommended in current guidelines [9], the efficacy of lifestyle intervention for NAFLD is often met with skepticism, especially with regard to its long-term outcomes [14]. This is owing to the fact that obesity in itself is a chronic disease, and weight loss can be difficult to achieve and maintain. Unfortunately, this often leads to a certain degree of therapeutic nihilism in management of these patients.

Our study demonstrated a high rate of sustained weight loss (mean weight loss of 20.3% and 22.9% in females and males, respectively) in mostly morbidly obese participants. The observational design of our study is a strength in this regard, as it was reflective of the real-world setting in which participants were treated for obesity as part of their routine clinical care.

To date, evidence to support a therapeutic effect of weight loss through lifestyle intervention for NAFLD is lacking in morbidly obese patients. The effect of lifestyle intervention has only been established in overweight cohorts [10–13]. Since the prevalence of NAFLD is highest in morbidly obese patients [33], our study focused on the most relevant target group. Our data suggest that weight loss and lifestyle changes are associated with significant improvements in the likelihood of both NASH and liver fibrosis, each determined by two independent non-invasive measures in morbidly obese patients. The data presented here are in line with one previous study [34]. That study had observed patients undergoing an industry-dependent lifestyle intervention program and reported an improvement of NFS scores in a small group of patients (*n* = 43). Here, we add data on the likelihood of liver inflammation/NASH and, more importantly, observed a larger cohort.

Another previous trial had histologically demonstrated an association of weight loss by lifestyle intervention with improvements in liver fibrosis [10]. However, that study had suffered from low response rates in terms of weight loss (9.9% of participants had achieved a weight loss > 10%), and it had focused on overweight rather than obese patients, two limitations which gave the rationale for this current study.

This current study, too, has important limitations. The lack of histology, elastography, or radiological assessment of liver parenchyma to detect or quantify fibrosis is its most important limitation. While clearly the gold standard is histological assessment, obtaining liver biopsies is only feasible in clinical studies. They, in turn, place higher enrollment obstacles and many participants refrain from taking part in those studies, owing to the increased risk associated with taking liver biopsies in morbidly obese patients. Thus, to obtain data from a real-world setting and to allow for repeated assessments, the assessment of both NASH and liver fibrosis must rely on non-invasive markers, as acknowledged and recommended by current guidelines [9, 15]. Obviously, each marker used in this study has its strengths and weaknesses. In particular, the NFS score is better at ruling out rather than detecting advanced fibrosis [9, 15, 18, 35, 36]. In one meta-analysis, its negative predictive value (NPV) for advanced fibrosis was 91.8% (cut-off − 1.455), while its positive predictive value was 66.9% (cut-off 0.676) with an overall AUROC of 0.84 [35]. Furthermore, except for FILI, the surrogate markers used were not originally intended to pick up changes in fibrosis in longitudinal studies. One multicenter longitudinal assessment of NFS confirmed its good AUROC for the detection of advanced fibrosis (0.78), but AUROC was reduced to 0.66 in the longitudinal application to detect changes in fibrosis, specifically in that study, progression in fibrosis [37]. Improvements in fibrosis were not seen, thus no AUROC for improved fibrosis could be calculated.

Accuracy of non-invasive tests is furthermore influenced by pre-test probabilities in the cohorts tested. While the FLI was developed for the detection of fatty liver in the general population [16], the NFS was established in selected patient cohorts with known NAFLD and in cohorts with high prevalence of liver fibrosis [18]. Although recommended for use as a screening tool in patients with obesity [15], the accuracy of FLI and NFS has not been rigorously tested in morbidly obese and may, therefore, be reduced in our cohort. The effect of pre-test probabilities may be particularly important for the interpretation of the apoptosis marker M30. While a cut-off of 200U/L has been repeatedly used to detect NASH or advanced liver disease [19, 29–31], its predictive value remains subject to further evaluation.

Our results must, therefore, be interpreted with caution and future studies will need to include elastographic or radiological assessments of liver parenchyma to validate our results. To address the limitations of surrogate scores, two independent non-invasive measures were applied for each entity. In addition to ALT, M30 and C16:1n7 were determined to reflect the course of liver inflammation, i.e. NASH, and both NFS and FILI were calculated to assess improvements in liver fibrosis.

Our study was single-armed, which is another limitation of the study. Nevertheless, all patients had undertaken multiple futile attempts to lose body weight previously, so spontaneous improvements without intervention would have been unlikely.

Importantly, 73% of participants were female and gender might influence the likelihood of adherence to lifestyle intervention. There is insufficient data to rule out this possible source of bias; however, available evidence suggests that males are not less adherent than women [38]. Given the paucity of structured lifestyle intervention programs, our cohort may furthermore have been comprised of particularly motivated obese patients and effectiveness of lifestyle intervention in terms of weight loss might be different when applied to a broader population.

Specific minimal weight loss thresholds required to improve NAFLD have been suggested. While some studies found that ≥ 7% weight loss was required for histological improvement in NASH [12, 39], others found ≥ 10% weight loss to be the minimal threshold [10]. Since 93% of participants in our study lost ≥ 10% of weight, there was an insufficient number of patients with weight loss < 10% to revisit such thresholds. Instead, we found a linear correlation of ALT improvement with weight loss, albeit with a low slope of the regression curve (supplementary Fig. 2). This suggests that any amount of weight loss may be beneficial to improve liver damage. Since virtually all subjects included in this study were still overweight at the end of the program (minimal BMI remaining > 25 kg/m^2^ in all but four participants at the end of treatment), no upper limit of a benefit by weight loss could be detected.

Only few structured weight loss programs are available for obese patients, even in industrialized countries. This may partly be due to an underestimation of the clinical need, efficacy and cost-effectiveness of such programs by caregivers, patients and health insurance companies alike. In the treatment of this chronic disease, keeping the weight off and developing healthy habits after the initial weight loss phase is a very important goal of any lifestyle program. Currently, patients and caregivers greatly rely on commercially available programs, which focus on dietary intervention, i.e. formula diets focused on short-term effects, but lack continuous long-term psychosocial counselling, physical training and nutritional consultation to prevent weight cycling and to support a sustained and long-lasting weight loss. Unfortunately, rigid commercial regimens resist adaptation due to licensing issues. We, therefore, developed this industry-independent lifestyle intervention program based on long-term experiences from our multiprofessional team. It is noteworthy to mention the relatively low costs of multimodal lifestyle intervention of this industry-independent program. Despite the intensive treatment regimen applied here, lifestyle intervention could be offered at moderate cost of 2,800 € per year.

In summary, our study adds to the growing body of evidence suggesting efficacy of lifestyle intervention to improve NAFLD in morbidly obese patients and demonstrates applicability of multimodal lifestyle intervention in this difficult to treat group of patients in a real-world setting. Our study thus encourages a better and more widespread application of this viable treatment option, and provides promising treatment results defying therapeutic nihilism.

## Supplementary Information

Below is the link to the electronic supplementary material.

Supplementary file1 (PPTX 68 KB)
