# Supplementary material for: Multidisciplinary lifestyle intervention is associated with improvements in liver damage and in surrogate scores of NAFLD and liver fibrosis in morbidly obese patients
Source: Eur J Nutr. 2022 Mar 11;61(5):2725–35. doi: 10.1007/s00394-022-02846-7 (PMC9279260; doi:10.1007/s00394-022-02846-7)
Supplement: Supplementary file 1 — Supplementary file1 (PPTX 68 KB) [file 394_2022_2846_MOESM1_ESM.pptx]

## Slide 1
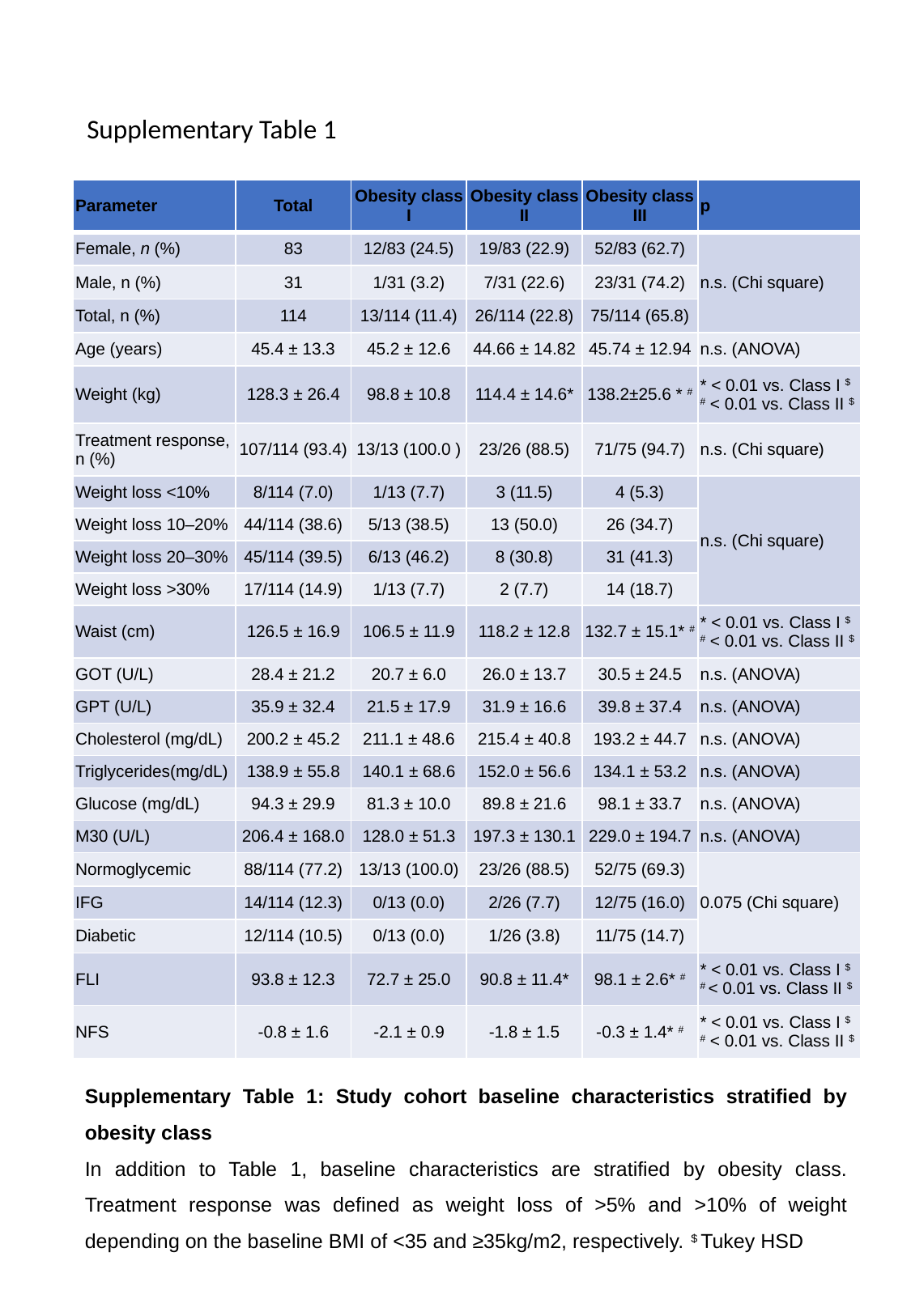

Supplementary Table 1
| Parameter | Total | Obesity class I | Obesity class II | Obesity class III | p |
| --- | --- | --- | --- | --- | --- |
| Female, n (%) | 83 | 12/83 (24.5) | 19/83 (22.9) | 52/83 (62.7) | n.s. (Chi square) |
| Male, n (%) | 31 | 1/31 (3.2) | 7/31 (22.6) | 23/31 (74.2) | |
| Total, n (%) | 114 | 13/114 (11.4) | 26/114 (22.8) | 75/114 (65.8) | |
| Age (years) | 45.4 ± 13.3 | 45.2 ± 12.6 | 44.66 ± 14.82 | 45.74 ± 12.94 | n.s. (ANOVA) |
| Weight (kg) | 128.3 ± 26.4 | 98.8 ± 10.8 | 114.4 ± 14.6\* | 138.2±25.6 \* # | \* < 0.01 vs. Class I $ # < 0.01 vs. Class II $ |
| Treatment response, n (%) | 107/114 (93.4) | 13/13 (100.0 ) | 23/26 (88.5) | 71/75 (94.7) | n.s. (Chi square) |
| Weight loss <10% | 8/114 (7.0) | 1/13 (7.7) | 3 (11.5) | 4 (5.3) | n.s. (Chi square) |
| Weight loss 10–20% | 44/114 (38.6) | 5/13 (38.5) | 13 (50.0) | 26 (34.7) | |
| Weight loss 20–30% | 45/114 (39.5) | 6/13 (46.2) | 8 (30.8) | 31 (41.3) | |
| Weight loss >30% | 17/114 (14.9) | 1/13 (7.7) | 2 (7.7) | 14 (18.7) | |
| Waist (cm) | 126.5 ± 16.9 | 106.5 ± 11.9 | 118.2 ± 12.8 | 132.7 ± 15.1\* # | \* < 0.01 vs. Class I $ # < 0.01 vs. Class II $ |
| GOT (U/L) | 28.4 ± 21.2 | 20.7 ± 6.0 | 26.0 ± 13.7 | 30.5 ± 24.5 | n.s. (ANOVA) |
| GPT (U/L) | 35.9 ± 32.4 | 21.5 ± 17.9 | 31.9 ± 16.6 | 39.8 ± 37.4 | n.s. (ANOVA) |
| Cholesterol (mg/dL) | 200.2 ± 45.2 | 211.1 ± 48.6 | 215.4 ± 40.8 | 193.2 ± 44.7 | n.s. (ANOVA) |
| Triglycerides(mg/dL) | 138.9 ± 55.8 | 140.1 ± 68.6 | 152.0 ± 56.6 | 134.1 ± 53.2 | n.s. (ANOVA) |
| Glucose (mg/dL) | 94.3 ± 29.9 | 81.3 ± 10.0 | 89.8 ± 21.6 | 98.1 ± 33.7 | n.s. (ANOVA) |
| M30 (U/L) | 206.4 ± 168.0 | 128.0 ± 51.3 | 197.3 ± 130.1 | 229.0 ± 194.7 | n.s. (ANOVA) |
| Normoglycemic | 88/114 (77.2) | 13/13 (100.0) | 23/26 (88.5) | 52/75 (69.3) | 0.075 (Chi square) |
| IFG | 14/114 (12.3) | 0/13 (0.0) | 2/26 (7.7) | 12/75 (16.0) | |
| Diabetic | 12/114 (10.5) | 0/13 (0.0) | 1/26 (3.8) | 11/75 (14.7) | |
| FLI | 93.8 ± 12.3 | 72.7 ± 25.0 | 90.8 ± 11.4\* | 98.1 ± 2.6\* # | \* < 0.01 vs. Class I $ # < 0.01 vs. Class II $ |
| NFS | -0.8 ± 1.6 | -2.1 ± 0.9 | -1.8 ± 1.5 | -0.3 ± 1.4\* # | \* < 0.01 vs. Class I $ # < 0.01 vs. Class II $ |
Supplementary Table 1: Study cohort baseline characteristics stratified by obesity class
In addition to Table 1, baseline characteristics are stratified by obesity class. Treatment response was defined as weight loss of >5% and >10% of weight depending on the baseline BMI of <35 and ≥35kg/m2, respectively. $ Tukey HSD

## Slide 2
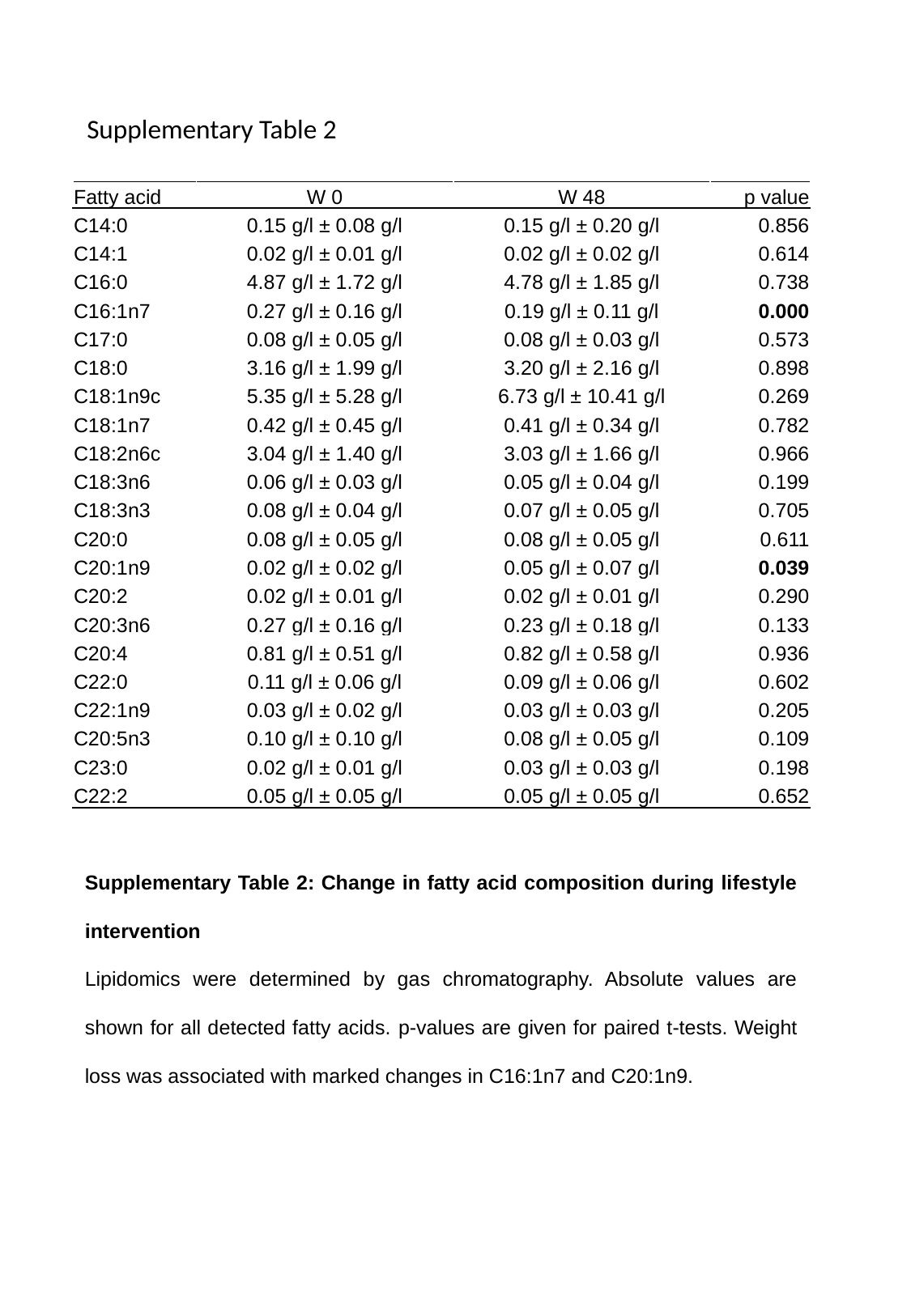

Supplementary Table 2
| Fatty acid | W 0 | W 48 | p value |
| --- | --- | --- | --- |
| C14:0 | 0.15 g/l ± 0.08 g/l | 0.15 g/l ± 0.20 g/l | 0.856 |
| C14:1 | 0.02 g/l ± 0.01 g/l | 0.02 g/l ± 0.02 g/l | 0.614 |
| C16:0 | 4.87 g/l ± 1.72 g/l | 4.78 g/l ± 1.85 g/l | 0.738 |
| C16:1n7 | 0.27 g/l ± 0.16 g/l | 0.19 g/l ± 0.11 g/l | 0.000 |
| C17:0 | 0.08 g/l ± 0.05 g/l | 0.08 g/l ± 0.03 g/l | 0.573 |
| C18:0 | 3.16 g/l ± 1.99 g/l | 3.20 g/l ± 2.16 g/l | 0.898 |
| C18:1n9c | 5.35 g/l ± 5.28 g/l | 6.73 g/l ± 10.41 g/l | 0.269 |
| C18:1n7 | 0.42 g/l ± 0.45 g/l | 0.41 g/l ± 0.34 g/l | 0.782 |
| C18:2n6c | 3.04 g/l ± 1.40 g/l | 3.03 g/l ± 1.66 g/l | 0.966 |
| C18:3n6 | 0.06 g/l ± 0.03 g/l | 0.05 g/l ± 0.04 g/l | 0.199 |
| C18:3n3 | 0.08 g/l ± 0.04 g/l | 0.07 g/l ± 0.05 g/l | 0.705 |
| C20:0 | 0.08 g/l ± 0.05 g/l | 0.08 g/l ± 0.05 g/l | 0.611 |
| C20:1n9 | 0.02 g/l ± 0.02 g/l | 0.05 g/l ± 0.07 g/l | 0.039 |
| C20:2 | 0.02 g/l ± 0.01 g/l | 0.02 g/l ± 0.01 g/l | 0.290 |
| C20:3n6 | 0.27 g/l ± 0.16 g/l | 0.23 g/l ± 0.18 g/l | 0.133 |
| C20:4 | 0.81 g/l ± 0.51 g/l | 0.82 g/l ± 0.58 g/l | 0.936 |
| C22:0 | 0.11 g/l ± 0.06 g/l | 0.09 g/l ± 0.06 g/l | 0.602 |
| C22:1n9 | 0.03 g/l ± 0.02 g/l | 0.03 g/l ± 0.03 g/l | 0.205 |
| C20:5n3 | 0.10 g/l ± 0.10 g/l | 0.08 g/l ± 0.05 g/l | 0.109 |
| C23:0 | 0.02 g/l ± 0.01 g/l | 0.03 g/l ± 0.03 g/l | 0.198 |
| C22:2 | 0.05 g/l ± 0.05 g/l | 0.05 g/l ± 0.05 g/l | 0.652 |
Supplementary Table 2: Change in fatty acid composition during lifestyle intervention
Lipidomics were determined by gas chromatography. Absolute values are shown for all detected fatty acids. p-values are given for paired t-tests. Weight loss was associated with marked changes in C16:1n7 and C20:1n9.

## Slide 3
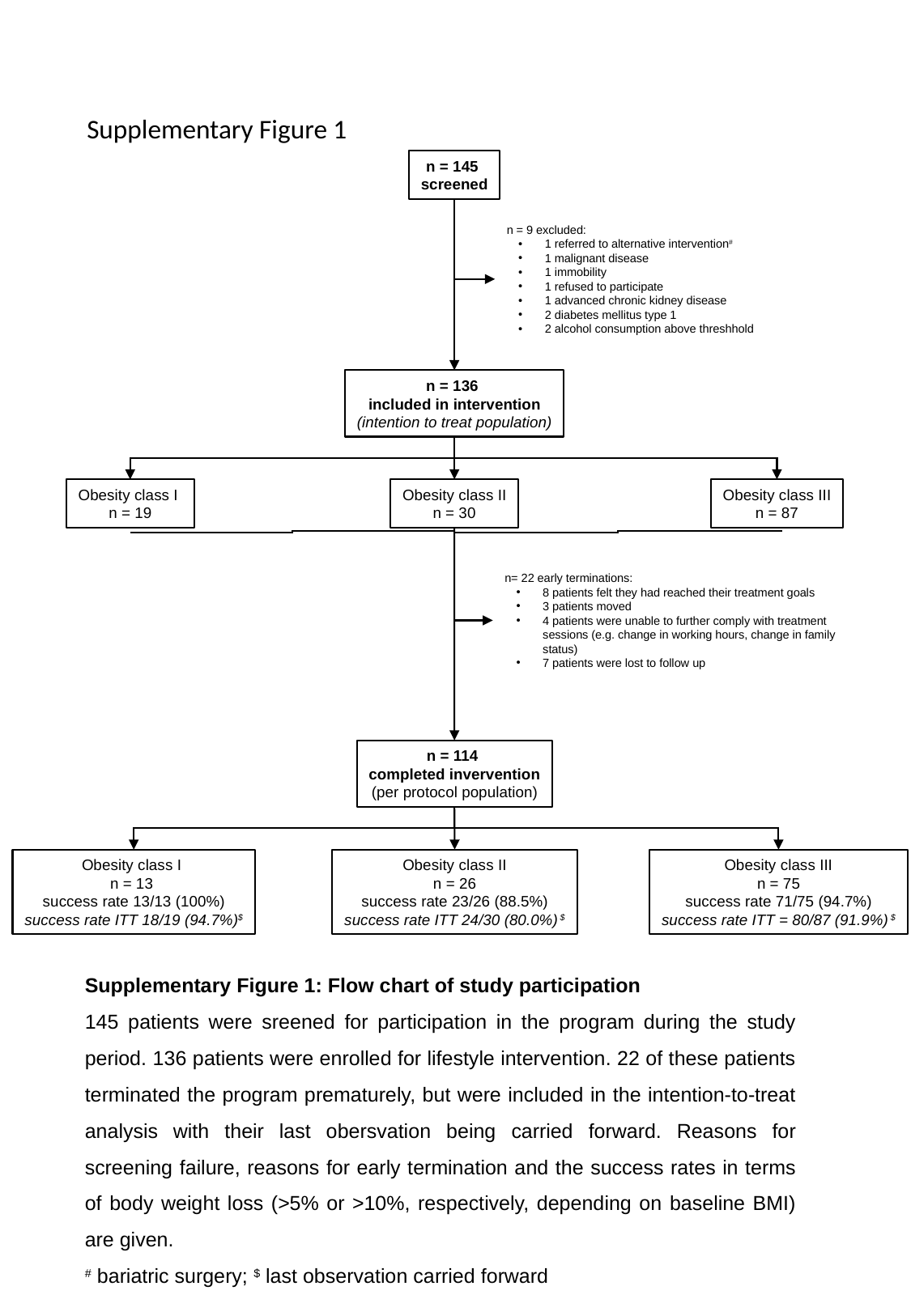

Supplementary Figure 1
n = 145
screened
n = 9 excluded:
1 referred to alternative intervention#
1 malignant disease
1 immobility
1 refused to participate
1 advanced chronic kidney disease
2 diabetes mellitus type 1
2 alcohol consumption above threshhold
n = 136
included in intervention
(intention to treat population)
Obesity class I
n = 19
Obesity class II
n = 30
Obesity class III
n = 87
n= 22 early terminations:
8 patients felt they had reached their treatment goals
3 patients moved
4 patients were unable to further comply with treatment sessions (e.g. change in working hours, change in family status)
7 patients were lost to follow up
n = 114
completed invervention
(per protocol population)
Obesity class I
n = 13
success rate 13/13 (100%)
success rate ITT 18/19 (94.7%)$
Obesity class II
n = 26
success rate 23/26 (88.5%)
success rate ITT 24/30 (80.0%) $
Obesity class III
n = 75
success rate 71/75 (94.7%)
success rate ITT = 80/87 (91.9%) $
Supplementary Figure 1: Flow chart of study participation
145 patients were sreened for participation in the program during the study period. 136 patients were enrolled for lifestyle intervention. 22 of these patients terminated the program prematurely, but were included in the intention-to-treat analysis with their last obersvation being carried forward. Reasons for screening failure, reasons for early termination and the success rates in terms of body weight loss (>5% or >10%, respectively, depending on baseline BMI) are given.
# bariatric surgery; $ last observation carried forward

## Slide 4
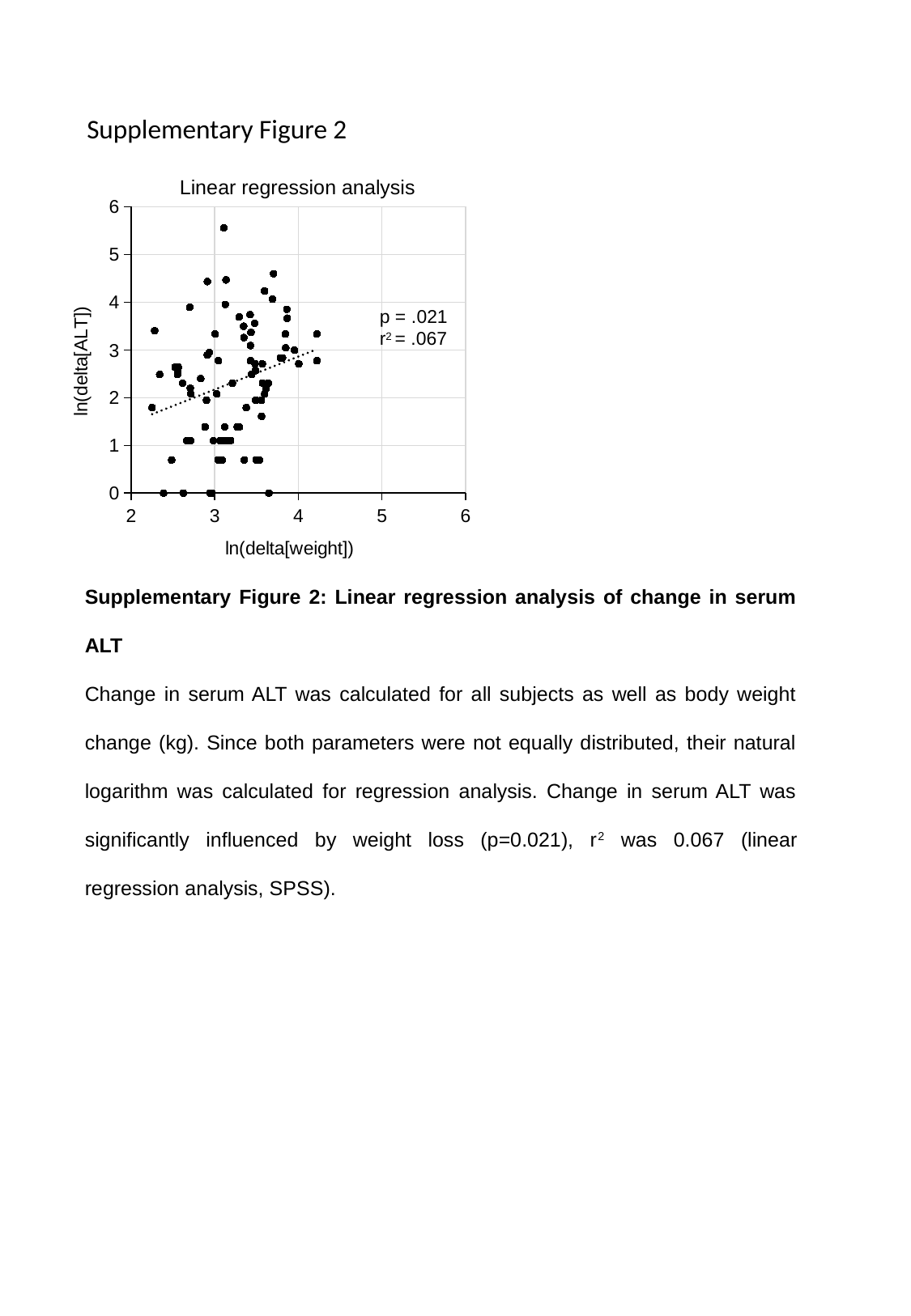

Supplementary Figure 2
Linear regression analysis
### Chart
| Category | |
|---|---|
p = .021
r2 = .067
Supplementary Figure 2: Linear regression analysis of change in serum ALT
Change in serum ALT was calculated for all subjects as well as body weight change (kg). Since both parameters were not equally distributed, their natural logarithm was calculated for regression analysis. Change in serum ALT was significantly influenced by weight loss (p=0.021), r2 was 0.067 (linear regression analysis, SPSS).
